# Tuberculosis and Silicosis Burden in Artisanal and Small-Scale Gold Miners in a Large Occupational Health Outreach Programme in Zimbabwe

**DOI:** 10.3390/ijerph182111031

**Published:** 2021-10-20

**Authors:** Dingani Moyo, Christopher Zishiri, Ronald Ncube, Godknows Madziva, Charles Sandy, Reginald Mhene, Nicholas Siziba, Fungai Kavenga, Florence Moyo, Orippa Muzvidziwa, Petronella Ncube, Blessings Chigaraza, Andrew Nyambo, Collins Timire

**Affiliations:** 1 Baines Occupational Health Services, Harare 024, Zimbabwe; drgmadziva@gmail.com (G.M.); flormoyo@iwayafrica.co.zw (F.M.); orippam@bainesohs.org (O.M.); petronellan@bainesohs.org (P.N.); blessingsc@bainesohs.org (B.C.); 2 Occupational Health Division, School of Public Health, University of the Witwatersrand, Johannesburg 2193, South Africa; 3 Department of Community Medicine, Faculty of Medicine, Midlands State University, Gweru 054, Zimbabwe; 4 Department of Community Medicine, Faculty of Medicine, National University of Science and Technology, Bulawayo 029, Zimbabwe; 5 The Union Zimbabwe Trust, Harare 024, Zimbabwe; rncube@uzt.org.zw; 6 Ministry of Health and Child Care, Harare 024, Zimbabwe; dr.c.sandy@gmail.com (C.S.); reginaldmhenedoc@gmail.com (R.M.); sitwalo@gmail.com (N.S.); drkav8@gmail.com (F.K.); andrewnyambo@gmail.com (A.N.); collinstimire2005@yahoo.com (C.T.)

**Keywords:** tuberculosis, silico-tuberculosis, silicosis, artisanal miners

## Abstract

Artisanal and small-scale miners (ASMs) labour under archaic working conditions and are exposed to high levels of silica dust. Exposure to silica dust has been associated with an increased risk of tuberculosis and silicosis. ASMs are highly mobile and operate in remote areas with near absent access to health services. The main purpose of this study was to evaluate the prevalence of tuberculosis, silicosis and silico-tuberculosis among ASMs in Zimbabwe. A cross-sectional study was conducted from 1 October to 31 January 2021 on a convenient sample of 514 self-selected ASMs. We report the results from among those ASMs who attended an outreach medical facility and an occupational health clinic. Data were collected from clinical records using a precoded data proforma. Data variables included demographic (age, sex), clinical details (HIV status, GeneXpert results, outcomes of chest radiographs, history of tuberculosis) and perceived exposure to mine dust. Of the 464 miners screened for silicosis, 52 (11.2%) were diagnosed with silicosis, while 17 (4.0%) of 422 ASMs were diagnosed with tuberculosis (TB). Of the 373 ASMs tested for HIV, 90 (23.5%) were sero-positive. An HIV infection was associated with a diagnosis of silicosis. There is need for a comprehensive occupational health service package, including TB and silicosis surveillance, for ASMs in Zimbabwe. These are preliminary and limited findings, needing confirmation by more comprehensive studies.

## 1. Introduction

Artisanal and small-scale gold mining (ASGM) occurs in about 80 countries, and there are more than 100 million artisanal and small-scale gold miners globally [1]. Within sub-Saharan Africa, informal employment is the main source of employment in central Africa (91.0%), eastern Africa (91.6%) and western Africa (92.4%) [2].

Worldwide, the incidence of silicosis is declining due to a combination of better engineering controls and other interventions to reduce industrial dust levels [3]. On the contrary, epidemiological studies show that 30–50% of workers may be suffering from silicosis and other pneumoconiosis in low- and middle-income countries [3]. The Southern African Development Community (SADC) recognised the triple epidemic of silicosis, tuberculosis (TB) and human immunodeficiency virus (HIV) infection in the large population of miners and ex-miners in southern Africa as needing urgent control across all member states [4]. Countries like Lesotho have extremely high prevalences of silicosis and silico-tuberculosis, 42.5% and 25.7%, respectively, among ex-miners [5]. For many years, Zimbabwe was among the top 30 high TB burdened countries [6].

Zimbabwe has an estimated population of more than 500,000 artisanal and small-scale miners (ASMs), with over one and a half million people depending on artisanal and small-scale mining [7]. In 2016, ASGM contributed 45% of the national gold production, contributing substantially to Zimbabwe’s revenue generation [8]. Artisanal and small-scale mining is characterised by excessive exposure to occupational hazards such as mercury- and silica-containing dust [9,10,11]. It is also associated with multiple health conditions such as respiratory, cardiovascular and musculoskeletal disorders, malaria, anaemia, diabetes mellitus and physical injuries [12]. Exposure to silica-containing dust is associated with an increased risk of developing silicosis and/or TB [13]. There is a well-documented increased risk of TB and fungal lung infections in patients with silicosis [13,14,15,16] and the risk of developing TB increases with an increasing severity of silicosis [17]. Silica dust has been classified as a group I carcinogen by the International Agency for Research on Cancer and is associated with lung cancer [18].

Silicosis is a permanent and irreversible parenchymal lung disease that results from the inhalation of silicon dioxide or silica in crystalline form [13]. Crystalline silica is a major component of rock and sand, and it is also found in soil, concrete, mortar, granite, other minerals and artificial stone [13,19]. Silica dust is intensely fibrotic, producing a characteristic lesion called the silicotic nodule in the lungs [20]. Inhaled silica dust is ingested by alveolar macrophages, which produce cytokines which stimulate fibroblasts to proliferate and produce increased amounts of collagen, resulting in lung fibrosis. However, the pathological changes in silica-injured lungs are complex and not completely understood [21]. In the early stages of silicosis, a chest radiograph shows isolated opacities against a normal lung parenchyma that can progress over years to a reticulonodular infiltrate. Simple silicosis can progress over several months or years to progressive massive fibrosis [20].

Artisanal miners labour under archaic and difficult working conditions and live in extreme poverty [22]. They also have poor health-seeking behaviours and live in overcrowded and poor shelters. Overcrowding and poorly ventilated shafts predispose them to both silicosis and TB. The highly nomadic life of ASMs complicates the provision of comprehensive health services. It may be difficult for this group to adhere to TB treatment since they must attend scheduled clinic visits for at least six months. The providers are not without challenges. One of the challenges is lack of occupational health expertise and services in most primary health care facilities in Zimbabwe [23,24]. It is very possible that many ASMs with silicosis and silica dust exposures, which are risk factors of TB, are being missed. In 2018, TB yields in ASMs attending an occupational health clinic in Zimbabwe were extremely high at 13% [25]. It is quite possible that the proportion of ASMs with silicosis is high. This is plausible because ASMs work in dusty environments with poor ventilation and without adequate personal protective equipment (PPE). There is currently a knowledge gap regarding the proportion of ASMs with silicosis, TB and silico-tuberculosis. The purpose of this study was to evaluate the burden of TB and silicosis among ASMs in Zimbabwe. The specific objectives were to: (i) Determine the proportion and characteristics of ASMs screened for and diagnosed with TB and silicosis; (ii) Assess the relationship between respiratory symptoms, chest X-ray and diagnostic yields of TB and silicosis; (iii) Evaluate the relationship between the duration of exposure to silica-containing dust and the diagnosis of silicosis.

## 2. Materials and Methods

### 2.1. The Study Design

This was a cross-sectional retrospective review of occupational health records of ASMs who volunteered to be screened for TB and silicosis at the occupational health clinic at Gweru Provincial Hospital and those consulted during a medical outreach facility at artisanal and small-scale mining sites in the Midlands and Matabeleland South provinces.

### 2.2. General Setting 

Zimbabwe is a landlocked country with 10 provinces and 64 administrative districts. In 2019, 60% of the notified TB cases in Zimbabwe were coinfected with HIV [26]. The Matabeleland South and Midlands provinces have high artisanal mining activities. Screening for silicosis and TB was conducted within the context of Zimbabwe’s Kunda-Nqob’ iTB project, funded through the United States Agency for International Development (USAID)’s TB Local Organization Network funding mechanism. The intervention targeted ASMs in the selected project districts of Zvishavane, Insiza, Gwanda and Gweru. Both males and females engage in the same gold mining activities. Therefore, a miner was defined as any person who was involved in the extraction and/or processing of gold. The outreach medical facility was provided at the ASGM mining sites, and all willing miners were free to attend. There was no prescreening of ASMs at either medical facility.

### 2.3. Specific Setting and Screening Procedure

All ASMs were assessed for TB using a symptoms screen tool and chest radiographs. The diagnosis of TB was based on a positive Cepheid GeneXpert test (Cepheid, SunnyVale, CA, USA) and/or clinical findings as per national TB Guidelines [26]. Screening for silicosis was done using chest radiographs. Interpretations of the chest radiographs were performed by medical officers trained in the diagnosis of occupational lung diseases (OLD) and TB, and were further checked by a specialist occupational physician trained and experienced in the International Labor Organisation (ILO) classification of chest radiographs. The diagnostic criteria for silicosis were a bilateral multinodular pattern with or without progressive massive fibrosis on a chest radiograph, a positive occupational history of exposure to silica-containing dust and having or not having symptoms of a subtle and progressive shortness of breath or a dry cough, in the absence of any other identifiable disease/s [13]. A diagnosis of silicosis was based on a threshold profusion of ≥1/0 on the ILO classification of chest radiographs [27]. The diagnosis of silico-TB was based on the presence of both silicosis and TB. Since the study was conducted during the COVID-19 pandemic, spirometry testing was not conducted as it is an aerosol-generating procedure. ASMs were captured in the presumptive TB register and the occupational health record. For the mobile outreaches, the presumptive TB registers used were from the nearest clinics within the catchment area where the outreaches were conducted. ASMs diagnosed with TB and/or silicosis were entered in the TB and TB-preventive therapy registers, respectively, and were linked to care through the local health facility.

### 2.4. Study Population

The source population consisted of ASMs working in the Midlands and Matabeleland South provinces in Zimbabwe. The study population consisted of ASMs working in the Midlands and Matabeleland South provinces from selected sites in Zvishavane, Gweru, Insiza and Gwanda who attended an outreach medical facility or the occupational health clinic at Gweru Provincial Hospital between 1 October 2020 and 31 January 2021. The ASMs included self-employed men and women working on an individual basis as well as those working in family groups, in partnerships, or as members of cooperatives.

### 2.5. Inclusion Criteria

All complete occupational health records of ASMs who were attended to at the occupational health clinic and medical outreach facility were included in the study. The records containing complete demographic details, routine observations and tests such as chest X-ray, HIV and GeneXpert test results were included in the study.

### 2.6. Exclusion Criteria

Occupational health records with missing chest radiograph results and demographic data were excluded from the study. The records of ASMs seen from outside the stipulated project districts were excluded from the study.

### 2.7. Sampling Procedure

All 514 occupational health records of the ASMs that were available during the study period were included in the study. This sample size was higher than the minimum sample size of 385 that was calculated using the OpenEpi software v3.03 (Dean and Sullivan, Atlanta, GA, USA) [28]. The calculation was based on the following assumptions: a prevalence of 50%, 95% confidence interval and a precision of 0.05.

### 2.8. Data Variables, Sources of Data and Data Collection

Individual level data were extracted from occupational health records of ASMs screened at the two medical facilities. The data variables included age, gender, HIV status, history of TB, respiratory symptoms, comorbidities (any of the following self-reported conditions: diabetes mellitus, chronic obstructive pulmonary disease, hypertension, asthma, cancer and chronic cough), exposure to silica dust, duration of mining, substance and alcohol use. The assessment of exposure to silica dust was subjective and based on whether the ASMs perceived themselves as being exposed to dust or not during mining. The sources of data were occupational health clinical records for ASMs. Data were collected using a pre-coded data proforma.

### 2.9. Data Analysis

Data were single entered in MS Excel and were exported to Stata v 13.0 (Stata Corporation, College Station, TX, USA) for cleaning and analysis [29]. Categorical variables were summarised using proportions, while continuous variables were summarised using means and standard deviations. The key outcome variables, silicosis diagnosis, silico-TB diagnosis and TB diagnosis, were dichotomous. The Chi-square test was used to test for associations between each of the independent and outcome variables. The modified Poisson regression model with robust variance estimators was used to assess the independent association of each characteristic with key outcome variables after adjusting for the following confounders: age, sex, duration of exposure to silica, comorbidities, respiratory symptoms, HIV status, previous history of TB, substance use and use of personal protective equipment. The associations were expressed as prevalence ratios (PRs) and adjusted PRs. The level of significance was set at 5% (*p* < 0.05).

## 3. Results

A total of 641 ASMs were reached during the study period. Of these, 127 records were excluded from the study. The final sample size was 514. The demographic and clinical characteristics of the 514 ASMs who were enrolled in the study are presented in Table 1. There was a predominance of males (85%). Of the 373 ASMs who were tested for HIV, 90 (23.5%) were HIV positive. The modal age category was 25–34 years, which constituted a third of the study population. The mean age (SD) was 37 years (12.7). Almost all ASMs were exposed to silica dust (95%), and just above a quarter (27%) had a duration of employment in artisanal and small-scale mining of at least 10 years. Just under two-thirds (61%) of ASMs did not report any respiratory symptoms.

The factors associated with silicosis diagnosis are presented in Table 2. There were 52 (11.2%) patients who were diagnosed with silicosis (95% CI: 8.6–14.4). ASMs who tested positive for HIV were 2.8 times more likely to be diagnosed with silicosis compared to those who tested negative for HIV, adjusted prevalence ratio—aPR = 2.79, 95% CI: (1.01–7.66). Those who reported comorbidities were two times more likely to be diagnosed with silicosis.

The factors associated with the development of silico-TB are presented in Table 3. Ten patients from the 52 who were diagnosed with silicosis had developed silico-TB. The prevalence of silico-TB was 2.2% (95% CI: 1.2–3.9). Having a positive HIV status, a previous history of TB and presence of any respiratory symptoms was associated with a silico-TB diagnosis. However, the associations were not significant after adjusting for the other variables.

The factors associated with the development of TB in ASMs are presented in Table 4. The prevalence of TB among ASMs was 4% (95% CI: 2.5–6.4). Of the 17 ASMs diagnosed with TB, nine were bacteriologically confirmed, two had pleural effusions and six were diagnosed on clinical grounds. The median [interquartile range (IQR)] duration of employment in ASMs who had abnormal chest radiographs was 8 years (IQR: 3.5–15.0). This was significantly higher than the median duration of 5 years (IQR: 1.0–8.0) of employment among ASMs who had normal chest radiographs, *p* < 0.001.

## 4. Discussion

We have studied a group of 514 ASMs in Zimbabwe during the period from 1 October 2020 to 31 January 2021. We report the results from those attending an outreach medical facility and an occupational health clinic which searched for TB and silicosis, and measured patients’ HIV status. We found a high prevalence of silicosis and TB, despite short periods of exposure to silica-containing dust. An HIV infection increased the risk of silicosis by three-fold.

The burden of silicosis and TB is well documented in formally employed miners and ex-miners, but there is a dearth of research and published data on the burden of TB, silicosis, or silico-TB in ASMs [30]. This is the first study to be conducted in Zimbabwe and in Africa among ASMs describing the burden of silicosis, silico-TB and TB. Apart from a study conducted in southern Brazil describing the prevalence of silicosis, we did not find comparable studies focusing on ASMs [31].

The prevalence of TB in ASMs in our study was almost 15 times higher than the national TB point prevalence of 275 per 100,000 in Zimbabwe [32]. Studies in Ghana and Malawi reported TB prevalences of 0.9% and 12% [33,34], respectively. However, both studies were not comparable to our study since they focused on formally employed miners and used random sampling techniques. 

The prevalence of silicosis observed in our study was higher than the prevalence ratio of 3.08 reported in ASMs in southern Brazil [31]. However, our study employed convenience sampling, whereas the Brazil study employed random sampling. This poses a challenge in making valid comparisons between the two studies. Most studies reporting the prevalence of silicosis and TB in Africa were on formally employed miners and/or ex-miners utilising random sampling methods. These studies, though not comparable to our study, show prevalences of silicosis ranging from 3.8% in South Africa [35] to 42.5% and 24.6% reported in former gold miners from Lesotho, 26.6–31% in Botswana and 22–36% in former miners in South Africa [5,36,37,38]. The quoted prevalences for miners in formal employment serve to highlight the differences between the unique and disadvantaged ASMs and miners in formal employment with better occupational health and safety services and working conditions. Our study differs from other studies conducted on formally employed miners and ex-miners reporting silicosis and TB prevalence in many aspects. Firstly, our study population was relatively young. Median ages reported by other studies ranged from 42 to 62 years. Secondly, the median duration of 4 years exposure to silica dust in our study was shorter than durations ranging from 19 to 42 years reported in previous studies [5,36,37,38]. Furthermore, unlike other studies which focused on miners and ex-miners who were mainly underground miners, our study focused on informal ASMs who were actively involved in predominantly surface mining activities.

The association between HIV infection and silicosis has not been described previously. This is one of the first studies that focuses on the burden of HIV in ASMs with silicosis. Here we found that just under a quarter of ASMs with silicosis were HIV positive. This is an important finding, especially in the context of high HIV prevalence in Zimbabwe and much higher sero-prevalence amongst ASMs in this study. HIV burden among ASMs is compounded by the fact that ASMs are hypermobile and operate in hard-to-reach areas where access to healthcare services is poor. However, the causal pathway between HIV and silicosis is not fully understood, and this finding needs further exploration.

The lack of association between respiratory symptoms and silicosis has been previously described [5]. This study confirms that silicosis can be diagnosed even in asymptomatic individuals who do not present with any respiratory symptoms.

Our study was not without limitations. Firstly, some variables had missing data and this could have reduced the power of the study to reach statistical significance in some variables. Secondly, our study focused on active ASMs and this might have inadvertently introduced survival bias since ASMs who died of silicosis were excluded from the study. Thirdly, we employed convenience sampling unlike most studies reporting the prevalence of silicosis, which use mostly random sampling for selecting study participants. Moreover, the study may have suffered from “healthy worker effect” as ASMs who were either sick during study periods or exited the ASMs pool due to ill health were excluded from the study. Sicker ASMs are more likely to have a higher burden of OLD than those who were finally enrolled in the study. Thus, the prevalence of TB and silicosis reported here is likely to be an underestimate. It would have been important to assess how use of respiratory PPE differs among previously treated TB patients and those who were never treated for TB, however, correct and consistent use of respiratory PPE was not captured as a variable in this study. Moreover, it would have been important to assess the pack-years of cigarette smoking in patients, however tobacco smoking was also not captured as a variable in the study. Furthermore, the ASMs selected in this study only underwent a symptom and temperature screen for COVID-19. This could have affected the number of TB cases diagnosed on clinical grounds as COVID-19 can present with similar clinical and pulmonary manifestations. We did not analyse the prevalence of TB by the different grades of silicosis (acute, accelerated, chronic simple silicosis or complicated silicosis).

Despite these limitations, our study serves as the first baseline study focusing on the burden of silicosis and TB in ASMs in Zimbabwe, and paves the way for future research in the artisanal and small-scale mining population. Our study has important policy considerations. Since silicosis can be diagnosed among asymptomatic and symptomatic ASMs, enhanced access to radiology services as a basic screening tool for ASMs is crucial. Given that ASMs visit public health institutions when they feel unwell, there is need to integrate occupational health into primary health care. This can be achieved through: (i) Developing guidelines for OLD (or through integrating OLD into TB and HIV guidelines); (ii) Capacitating health care workers to provide health education and screening services to this group.

This study has shown that silicosis and TB in ASMs are a huge problem in Zimbabwe and are affecting young people. People aged 25–34 years have a high prevalence of TB in Zimbabwe [6]. The synergistic relationship between HIV and silicosis may fuel the TB epidemic in Zimbabwe since both increase the risk of TB. ASMs who get sick usually travel to their hometowns where they may act as foci for community transmission of TB.

## 5. Conclusions

Although our cross-sectional findings must be considered preliminary, the prevalence of TB, silicosis and silico-TB among ASMs in Zimbabwe is very high. There is need to provide a comprehensive occupational health service package, including TB and silicosis surveillance, to ASMs in Zimbabwe. Interventions to reduce exposure to silica-containing dust through raising awareness on safer mining methods are needed. This calls for enhanced collaboration between the Zimbabwe Miners Federation, Ministry of Mines, Ministry of Labour and Social Services and the Ministry of Health and Child Care.

## Figures and Tables

**Table 1:** Demographic and characteristics of participants who were enrolled in a large occupational health outreach programme in Zimbabwe, 2020–2021.

Characteristic		Number	(%) ‡
Total		514	(100)
Sex	Male	435	(85)
	Female	71	(14)
	Unknown	8	(1)
Age Category (Years)	15–19	17	(3)
	20–24	61	(12)
	25–34	167	(32)
	35–44	132	(26)
	45+	129	(25)
	Unknown	8	(2)
	Mean (SD)	37.0 (12.7)	
HIV Status	Positive	90	(18)
	Negative	283	(55)
	Unknown	141	(27)
Previous TB Treatment	Yes	62	(12)
	No	442	(86)
	Unknown	10	(2)
Comorbidities †	Yes	163	(32)
	No	342	(67)
	Unknown	9	(1)
Any Respiratory Symptoms	Yes	75	(14)
	No	312	(61)
	Unknown	127	(25)
Marijuana and/or Dagga Use	Yes	143	(28)
	No	365	(71)
	Unknown	6	(1)
Alcohol Use	Yes	266	(52)
	No	242	(47)
	Unknown	6	(1)
Exposure to Silica-Containing Dust	No	17	(3)
	Yes	487	(95)
	Unknown	10	(2)
Duration of Employment (Years)	<10	352	(69)
	≥10	137	(27)
	Unknown	25	(4)
	Median (IQR)	4 (0.02–33)	

‡ = Column percentages. † = Any of the following self-reported conditions: diabetes mellitus, hypertension, chronic obstructive pulmonary disease, cancer, asthma and chronic chest pains. TB = tuberculosis.

**Table 2:** Factors associated with silicosis diagnosis among ASMs who were enrolled in a large occupational health outreach programme in Zimbabwe, 2020–2021.

Characteristic		Total	Diagnosed with Silicosis		PR 95% CI	aPR 95% CI
			Number	(%) ‡		
		464 *	52	(11.2)		
Sex (*n* = 456)	Male	395	48	(12.1)	3.71 (0.92–14.9)	2.02 (0.45–8.96)
	Female	61	2	(3.3)	Ref	Ref
Age Category (*n* = 451)	15–19	17	0	(0.0)	–	–
	20–24	54	6	(10.0)	Ref	Ref
	25–34	157	17	(10.8)	1.08 (0.45–2.61)	1.06 (0.28–4.01)
	35–44	113	17	(15.0)	1.50 (0.63–3.61)	1.35 (0.34–5.43)
	45+	110	12	(10.9)	1.09 (0.43–2.76)	0.58 (0.12–2.80)
HIV Status (*n* = 343)	Positive	73	17	(23.3)	2.25 (1.30–3.87) **	2.79 (1.01–7.66) **
	Negative	270	28	(10.4)	Ref	Ref
History of TB Treatment (*n* = 455)	Yes	42	4	(9.5)	0.94 (0.35–2.48)	0.59 (0.13–2.62)
	No	413	42	(10.1)	Ref	Ref
Comorbidities † (*n* = 456)	Yes	139	21	(15.1)	1.92 (1.11–3.30) **	0.97 (0.36–2.63)
	No	317	25	(7.9)	Ref	Ref
Any Respiratory Symptom (*n* = 367)	Yes	66	9	(13.5)	1.37 (0.68–2.74)	1.14 (0.43–3.01)
	No	301	30	(10.0)	Ref	Ref
Exposure to silica-containing dust (*n* = 455)	No	17	2	(11.8)	Ref	Ref
	Yes	438	46	(10.5)	0.89 (0.24–3.38)	–
Duration of Employment (Years) (*n* = 444)	<10	324	29	(9.0)	Ref	Ref
	≥10	120	18	(15.1)	1.69 (0.97–2.90)	1.40 (0.62–3.17)

PR = Prevalence ratio; aPR = Adjusted prevalence ratio; CI = Confidence interval; ‡ = Row percentages. * Records with missing data either in the outcome variable or in the predictor variable were not included in the analysis. ** = statistically significant; † = Any of the following self-reported conditions: diabetes mellitus, hypertension, chronic obstructive pulmonary disease, cancer, asthma and chronic chest pains.

**Table 3:** Factors associated with silico-TB diagnosis among ASMs who were enrolled in a large occupational health outreach programme in Zimbabwe, 2020–2021.

Characteristic		Total	Diagnosed with Silico-TB		PR 95% CI	aPR 95% CI
			Number	(%) ‡		
		464 *	10	(2.2)		
Sex (*n* = 456)	Male	395	10	(2.5)	–	–
	Female	61	0	(0.0)	Ref	Ref
Age (Years) (*n* = 451)	15–19	17	0	(0.0)	–	–
	20–24	60	0	(0.0)	–	–
	25–34	157	3	(1.9)	Ref	Ref
	35–44	113	7	(6.2)	3.24 (0.86–12.2)	
	45+	110	0	(0.0)	–	–
HIV Status (*n* = 343)	Positive	73	7	(9.5)	8.63 (2.29–32.6) **	3.90 (0.46–32.9)
	Negative	270	3	(1.1)	Ref	Ref
History of TB Treatment (*n* = 455)	Yes	42	3	(7.1)	4.21 (1.13–15.7) **	1.68 (0.14–19.4)
	No	413	7	(1.7)	Ref	Ref
Comorbidities † (*n* = 456)	Yes	139	6	(4.3)	3. 42 (0.98–11.93)	0.87 (0.05–16.2)
	No	317	4	(1.3)	Ref	Ref
Any Respiratory Symptoms (*n* = 464)	Yes	163	8	(4.9)	7.38 (1.59–34.4) **	3.00 (0.40–22.2)
	No	301	2	(0.7)	Ref	Ref
Marijuana and/or Dagga Use (*n* = 459)	Yes	130	4	(3.1)	1.69 (0.48–5.88)	4.41 (0.43–45.2)
	No	329	6	(1.8)	Ref	Ref
Alcohol Use (*n* = 459)	Yes	243	8	(3.3)	3.56 (0.76–16.56)	0.44 (0.02–10.1)
	No	216	2	(0.9)	Ref	Ref
Exposed to Silica-Containing Dust (*n* = 455)	No	17	0	(0.0)	Ref	Ref
	Yes	438	10	(2.3)	–	–
Duration of Employment (Years) (*n* = 444)	<10	324	6	(1.9)	Ref	Ref
	≥10	120	4	(3.3)	1.8 (0.52–6.27)	0.74 (0.10–5.52)

PR = Prevalence ratio; aPR = Adjusted prevalence ratio; CI = Confidence interval; ‡ = Row percentages. * Records with missing data either in the outcome variable or in the predictor variable were not included in the analysis. ** = statistically significant † = Any of the following self-reported conditions: diabetes mellitus, hypertension, chronic obstructive pulmonary disease, cancer, asthma and chronic chest pains.

**Table 4:** Factors associated with TB diagnosis among ASMs who were enrolled in a large occupational health outreach programme in Zimbabwe, 2020.

Characteristic		Total	Diagnosed with TB		PR 95% CI	aPR 95% CI
			*N*	(%) ‡		
		422 *	17	(4.0)		
Sex (*n* = 416)	Male	357	17	(4.8)	–	–
	Female	59	0	(0.0)	Ref	Ref
Age Group (Years) (*n* = 415)	15–19	17	0	(0.0)	–	–
	20–24	54	0	(0.0)	–	–
	25–34	143	6	(4.2)	Ref	Ref
	35–44	103	8	(7.8)	1.35 (0.30–6.01)	1.09 (0.20–6.01)
	45+	98	2	(2.0)	0.48 (0.10–2.36)	1.10 (0.89–14.0)
HIV Status (*n* = 308)	Positive	63	9	(14.3)	4.40 (1.76–10.8) **	3.90 (0.35–43.0)
	Negative	245	8	(3.3)	Ref	Ref
History of TB Treatment (*n* = 419)	Yes	41	5	(12.2)	3.84 (1.42–10.4) **	1.12 (0.10–13.1)
	No	378	12	(3.2)	Ref	Ref
Comorbidities † (*n* = 420)	Yes	124	8	(6.5)	2.12 (0.84–5.37)	0.64 (0.02–9.60)
	No	296	9	(3.0)	Ref	Ref
Any Respiratory Symptoms (*n* = 332)	Yes	59	3	(5.1)	6.94 (1.19–40.6) **	4.07 (0.44–37.9)
	No	273	2	(0.1)	Ref	Ref
Marijuana and/or Dagga Use (*n* = 421)	Yes	118	6	(5.1)	1.40 (0.53–3.70)	15.3 (0.37–44.30)
	No	303	11	(3.6)	Ref	Ref
Alcohol Use (*n* = 421)	Yes	221	13	(5.9)	2.94 (0.97–8.87)	0.38 (0.01–14.7)
	No	200	4	(2.0)	Ref	Ref
Duration of Employment (Years) (*n* = 406)	<10	301	10	(3.3)	Ref	Ref
	≥10	105	7	(6.7)	1.63 (0.90–2.96)	2.69 (0.44–16.4)

* Records with missing data either in the outcome variable or in the predictor variable were not included in the analysis. ** = statistically significant; PR = Prevalence ratio; aPR = Adjusted prevalence ratio; CI = Confidence interval; ‡ = Row percentages. † = Any of the following self-reported conditions: diabetes mellitus, hypertension, chronic obstructive pulmonary disease, cancer, asthma and chronic chest pains.

## Data Availability

The data presented in this study are available on request from the corresponding author. The data are not publicly available due to authorisations that may be required by the Ministry of Health and Child Care in Zimbabwe.
